# Genomic analysis of a riboflavin-overproducing *Ashbya gossypii* mutant isolated by disparity mutagenesis

**DOI:** 10.1186/s12864-020-6709-7

**Published:** 2020-04-23

**Authors:** Tatsuya Kato, Junya Azegami, Ami Yokomori, Hideo Dohra, Hesham A. El Enshasy, Enoch Y. Park

**Affiliations:** 1 Green Chemistry Research Division, Research Institute of Green Science and Technology, Shizuoka University, Ohya 836, Suruga-ku, Shizuoka, Japan; 2 Department of Agriculture, Graduate School of Integrated Science and Technology, Shizuoka University, Ohya 836, Suruga-ku, Shizuoka, Japan; 3 Instrumental Research Support Office, Research Institute of Green Science and Technology, Shizuoka University, Ohya 836, Suruga-ku, Shizuoka, Japan; 4 Institute of Bioproduct Development (IBD), Universiti Teknologi Malaysia (UTM), 81310 UTM, Johor Bahru, Malaysia

**Keywords:** *Ashbya gossypii*, Riboflavin production, Disparity mutagenesis, Homozygous mutation, Heterozygous mutation

## Abstract

**Background:**

*Ashbya gossypii* naturally overproduces riboflavin and has been utilized for industrial riboflavin production. To improve riboflavin production, various approaches have been developed. In this study, to investigate the change in metabolism of a riboflavin-overproducing mutant, namely, the W122032 strain (MT strain) that was isolated by disparity mutagenesis, genomic analysis was carried out.

**Results:**

In the genomic analysis, 33 homozygous and 1377 heterozygous mutations in the coding sequences of the genome of MT strain were detected. Among these heterozygous mutations, the proportion of mutated reads in each gene was different, ranging from 21 to 75%. These results suggest that the MT strain may contain multiple nuclei containing different mutations. We tried to isolate haploid spores from the MT strain to prove its ploidy, but this strain did not sporulate under the conditions tested. Heterozygous mutations detected in genes which are important for sporulation likely contribute to the sporulation deficiency of the MT strain. Homozygous and heterozygous mutations were found in genes encoding enzymes involved in amino acid metabolism, the TCA cycle, purine and pyrimidine nucleotide metabolism and the DNA mismatch repair system. One homozygous mutation in *AgILV2 gene* encoding acetohydroxyacid synthase, which is also a flavoprotein in mitochondria, was found. Gene ontology (GO) enrichment analysis showed heterozygous mutations in all 22 DNA helicase genes and genes involved in oxidation-reduction process.

**Conclusion:**

This study suggests that oxidative stress and the aging of cells were involved in the riboflavin over-production in *A. gossypii* riboflavin over-producing mutant and provides new insights into riboflavin production in *A. gossypii* and the usefulness of disparity mutagenesis for the creation of new types of mutants for metabolic engineering.

## Background

*Ashbya gossypii,* a filamentous fungus, is a riboflavin producer and has been utilized for industrial riboflavin production. Therefore, many studies on the metabolic mechanism of riboflavin production in *A. gossypii* have been carried out, and several overproducing mutants have been isolated [1]. In addition, the genome of *A. gossypii* is very similar to that of *Saccharomyces cerevisiae*, which is a budding yeast, and 91% of 4476 annotated *A. gossypii* genes are syntenic to those of *S. cerevisiae* [2]. This finding provides for many researchers to identify differences between the growth of filamentous fungi and budding yeasts [3].

Isocitrate lyase (ICL), which catalyzes the cleavage reaction of isocitrate to succinate and glyoxylate, is an important enzyme for riboflavin production in *A. gossypii* [4]. The mutant isolated using itaconate, which is an ICL inhibitor, produced a 25-fold higher level of riboflavin in soybean oil-containing medium than the wild type. The mutant isolated on oxalate-containing medium showed a 5-fold higher riboflavin yield than wild type in rapeseed oil medium [5]. In addition, genetic engineering of this fungus has been utilized for riboflavin production [6]. Overexpression of riboflavin biosynthetic genes in *A. gossypii* contributed to the enhancement of riboflavin production [7]. Disruption of cytoplasmic serine hydroxymethyltransferase gene (*AgSHM2*) in *A. gossypii* also improved riboflavin production 10-fold compared to the wild type [8]. Reinforcement of the purine biosynthetic pathway in *A. gossypii* also improved riboflavin production [9, 10]. These results show that glycine and the purine biosynthetic pathway are important factors for riboflavin production in *A. gossypii*. Along with genetic engineering, metabolic investigation using a ^13^C tracer has been carried out to improve riboflavin production in *A. gossypii* [11, 12].

Recently, the *A. gossypii* w122032 mutant (MT strain), which is an overproducer of riboflavin, was isolated by the disparity mutagenesis method [13]. This disparity mutagenesis was first demonstrated by Furusawa et al., and disparity theory has been developed by computer simulation [14, 15]. Expression of error-prone DNA polymerase δ in hosts generates increased diversity of hosts that have mutated genomes and leads to the isolation of mutant strains with desired properties. In the MT strain, mutation sites in metabolic pathways were suggested by DNA microarray analysis, proteome analysis and metabolic flux analysis [13, 16]. However, definite mutation sites have not been identified to date.

In this study, using a next-generation DNA sequencer, genome analysis of the MT strain was carried out, and mutation sites in the genome of this mutant compared to that of wild type were determined to clarify the mechanism of the riboflavin over-production in MT strain considering the previous analyses of MT strain [13, 16]. In addition, we discussed the roles of genes mutated in the MT strain.

## Results and discussion

### Genome analysis of each strain and identification of mutations in the genome sequence of MT

We previously reported that the riboflavin over-producing mutant (MT strain) was isolated by disparity mutagenesis in the presence of H_2_O_2_, itaconate and oxalate and phenotypes of this MT strain were characterized by transcriptomic, proteomic and metabolic flux analyses [13, 16]. In this study, to reveal the genotype of MT strain, genome resequencing and single-nucleotide polymorphisms (SNP) analysis were carried out. Whole-genome shotgun sequencing for WT and MT generated 1,083,909 and 1,519,777 high-quality read pairs totaling approximately 593 and 836 Mb, respectively. The high-quality reads of WT and MT were aligned to the reference genome of *A. gossypii* ATCC10895, resulting in sequence coverages of 41.9–43.4 and 46.7–53.6, respectively, for chromosome I–VII. Among the variants identified by the Genome Analysis Toolkit (GATK) based on the aligned reads for WT and MT, mutations in open reading frames (ORFs), missense mutations, frameshift mutations and nonsense mutations were analyzed. In WT, which is same as the original strain *A. gossypii* ATCC10895, amino acid sequences encoded by all ORFs were the same as those of strain ATCC10895, except for the *SEN2* gene (AGOS_AGR073C), which encodes a subunit of the tRNA splicing endonuclease in *S. cerevisiae* (Supplementary material Table S1). This result indicates that this WT, which has been maintained in our laboratory, could have gained this heterozygous mutation. However, this WT was used in this study because this gene may not be involved in riboflavin production, given the function of the gene product. Additionally, some silent mutations were also detected (data not shown).

**Fig. 1:**
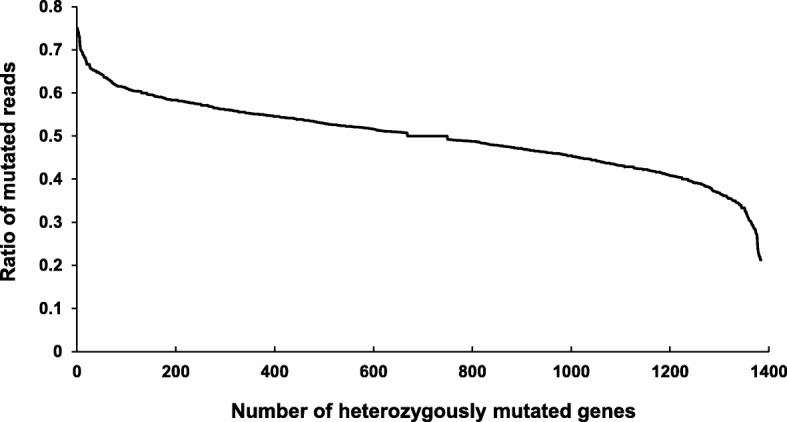
Proportion of mutated reads in each gene among 1377 heterozygous mutations in the coding sequences of the MT genome. The highest proportion was 75% (OCT1, AGOS_AFR198W), and the lowest proportion was 21% (AGOS_AGL272C). Most heterozygous mutations were detected at 40–60%

From the single-nucleotide variant (SNV) analysis between the genome sequences of WT and MT, we detected 33 homozygous and 1377 heterozygous mutations in the coding sequences of the genome of MT strain (Supplementary materials Tables S1 and S2), which cause missense, nonsense and frameshift mutations, in addition to silent mutations. These heterozygous mutations suggest that nuclei of the MT strain are polyploid. In the 1377 heterozygous mutations, the proportion of mutations in each gene was different. The highest proportion was 75% (chromosome VI:799,900 in *AgOCT1*, AGOS_AFR198W), and the lowest proportion was 21% (chromosome VII:198,537 and 198541 in *AgATP1*, AGOS_AGL272C) (Fig. 1). Most heterozygous mutants were found to have ratios of 40–60%. These results suggest that the MT strain may contain multiple nuclei containing different mutations. To prove its ploidy, we tried to isolate haploid spores from the MT strain, but this strain did not produce spores under the conditions tested. This result indicates that the MT strain lost the ability to sporulate even though it was previously reported that the riboflavin production in *A. gossypii* is related with its spore production [17]. *A. gossypii* is a naturally multinucleate fungus, but this fungus may be haploid, and the spores of this fungus produced by asexual sporulation are also haploid [2, 18]. However, Anderson et al. reported that ploidy variation was observed in *A. gossypii* with minor aneuploidy [19]. In this study, the proportion of heterozygous mutations in each gene ranged from 75 to 21%, and most heterozygous mutations were found at 40–60%. This result may be caused by the polyploidy or multinucleate cells of this organism. Anderson et al. [19] also discussed the low germination frequency of spores produced from variable polypoid nuclei. Two possibilities were suggested: a reduction in ploidy to uninucleate haploid spores and the formation of spores with variable ploidy. In this study, the MT strain never produced haploid spores.

**Fig. 2:**
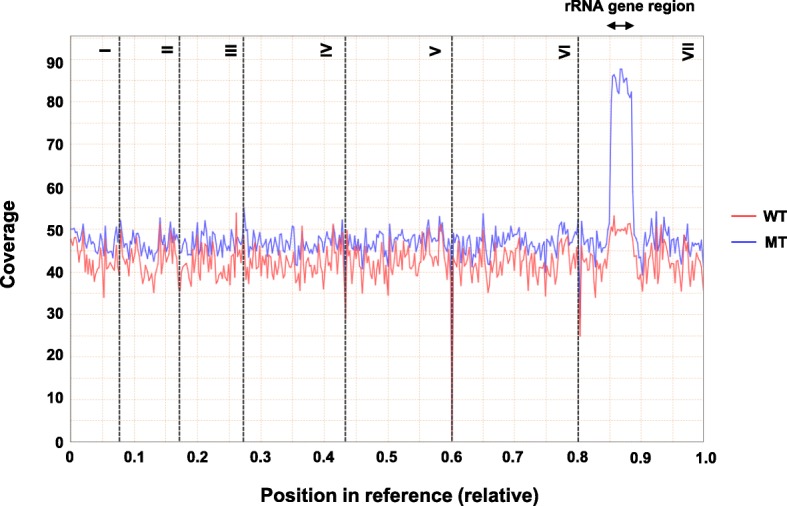
Sequence coverage line graph of chromosomes in MT strain and WT strain. Compared to the WT strain, a large number of rRNA gene repeat sequences in chromosome VII were detected in the MT strain

Which corresponds, interestingly, we found a region representing ~ 2-fold sequence coverage compared to other regions in chromosome VII of the MT strain, which correspond to the rRNA gene repeats (Chr VII:441,317-762,344) (Fig. 2). In yeasts, the number of rRNA gene repeats is normally maintained for genome stability and determination of life span [20, 21]. Moreover, the rRNA gene controls chromosome homeostasis [22]. When the number of rRNA gene repeats increases, rRNA gene instability and aging phenotypes are observed. Silva et al. showed that the riboflavin-overproducing *Ashbya* mutants are vulnerable to photoinduced oxidative DNA damage and accumulate reactive oxygen species (ROS) [23]. The ROS is largely involved in the aging of cells, suggesting that the riboflavin production in *A. gossypii* may be associated with the aging of cells.

It is reasonable that homozygous mutations have more crucial effects on riboflavin production in the MT strain compared to heterozygous mutations. We selected candidate mutations among 33 homozygous mutations in the coding sequence of the genome of MT strain, as shown in Table 1. Among the 33 homozygous mutations, the *SEN2* gene (AGOS_AGR073C) has one homozygous mutation in the MT strain, in contrast to the WT strain used in this study, which has one heterozygous mutation at the same nucleotide. Four homozygous mutations in the amino acid metabolism of *A. gossypii* were detected.

**Table 1:** Homozygous mutations of genes in MT strain

Chromosome	Position	WT seq.	MT seq.	Quality	Mutation	Gene	Product	DNA changes	Protein changes	Number	
										WT seq.	MT seq.
II	496,139	C	T	1495.42	missense	AGOS_ABR055C	Transcriptional activator (AgSOK2 or AgPHD1)	c.1180G > A	G394R	0	38
III	726,948	CG	C	1167.38	frameshift	AGOS_ACR215C	Cytosolic serine hydroxymethyltransferase (AgSHM2)	c.1332delC	p.Q445fs	0	30
IV	1,433,004	T	A	1442.42	missense	AGOS_ADR404C	Oleate-activated transcription factor (AgOAF1 or AgPIP2)	c.2317A > T	p.T773S	0	38
IV	1,433,040	T	G	1455.42	missense	AGOS_ADR404C	Oleate-activated transcription factor (AgOAF1 or AgPIP2)	c.2281A > C	p.T761P	0	39
IV	199,365	G	A	1523.42	missense	AGOS_ADL287C	Chorismate synthase (AgARO2)^a^	c.206C > T	p.T69M	0	39
V	70,024	C	A	1836.42	missense	AGOS_AEL305C	Large subunit of acetohydroxyacid synthase (AgILV2)^a^	c.1365G > T	p.Q455H	0	46
VII	791,717	C	A	1505.42	missense	AGOS_AGL123W	Cytidine deaminase (AgCDD1)	c.314C > A	p.P105Q	0	41
VII	962,069	G	A	1560.42	nonsense	AGOS_AGL036C	Heat shock protein 104 (AgHSP104)	c.1066C > T	p.Q356*	0	42
VI	1,753,850	G	A	1884.42	missense	AGOS_AGR382W	L-aminoadipate-semialdehyde dehydrogenase-phosphopantetheinyl transferase (AgLYS5)	c.365G > A	p.R122H	0	49

These homozygous mutations are a subset among all 32 homozygous mutations which are shown in Table S1

^a^Flavoproteins

*Translation stops here

First, a frameshift mutation in the *AgSHM2* gene (AGOS_ACR215C) was detected in the genome of the MT strain. This gene encodes serine hydroxymethyltransferase 2 (SHMT), and it was previously reported that disruption of this gene enhanced the productivity of riboflavin in *A. gossypii*, although the growth of the organism was compromised [7]. The frameshift mutation causes the deletion of 25 amino acid residues at the C-terminus of AgSHM2 and the addition of 6 extra amino acid residues in the deletion mutant. This C-terminal region may not be directly involved in catalytic activity [24]. However, the L474F mutation in this region of human and rabbit SHMT causes a decrease in the binding of this protein to co-factors [25]. Therefore, this frameshift mutation in the MT strain may lead to a decrease in the SHMT activity of AgSHM2. In addition to the homozygous frameshift mutation, one heterozygous mutation (593G → A), which causes a missense mutation, R198Q, was also detected in the *AgSHM2* gene.

Second, a missense mutation (206C → T) in the *AgARO2* gene (AGOS_ADL287C), which produces the T69M mutant, was detected. In *S. cerevisiae*, this gene encodes chorismate synthase, which produces chorismate, a building block of aromatic compounds. Because T69 in the chorismate synthase of *S. cerevisiae* is distant from the catalytic site, this residue may not be directly involved in catalytic activity [26]. In addition, this enzyme also exhibits flavin reductase activity for the synthesis of reduced flavin mononucleotide (FMN), which is required for chorismate synthase activity.

Third, a missense mutation (1365G → T) in the *AgILV2* gene (AGOS_AEL305C), which produces the Q455H mutant, was detected. In *S. cerevisiae*, this gene encodes the large subunit of acetohydroxyacid synthase (AHAS), which solely catalyzes the synthesis of 2-acetolactate and 2-aceto-2-hydroxybutyrate. This reaction is the first step of branched-chain amino acid biosynthesis. This mutation may not have considerable effects on enzymatic activity because Q455 is not in the co-factor-binding sites [27]. This enzyme requires flavin adenine dinucleotide (FAD) as a co-factor, even though this reaction does not require oxidation and reduction. A small subunit of AHAS encoded by the *ScILV6* gene regulates the AHAS activity of ScILV2 in yeast [28]. *A. gossypii* also has *AgILV2* and *AgILV6* genes. In *AgILV6* genes, three heterozygous missense mutations (140G → A, S47N; 155G → A, S52N; 673G → T, G225C) were detected.

Fourth, a missense mutation (365G → A) in the *AgLYS5* gene (AGOS_AGR382W), which produces the R122H mutant, was detected. In *S. cerevisiae*, ScLYS5 (4′-phosphopantetheinyl transferase, PPTase) converts the apo-form of ScLYS2 (α-aminoadipate reductase) to the active holo-form by the transfer of phosphopantetheine and is present in the lysine biosynthetic pathway [29]. In addition to modification, PPTase is involved in fungal growth, the biosynthesis of secondary metabolites and asexual and sexual development [30, 31].

In pyrimidine metabolism in *A. gossypii*, one homozygous mutation was detected in the *AgCDD1* gene (AGOS_AGL123W), which encodes cytosine deaminase in *S. cerevisiae*. This enzyme catalyzes the conversion of cytidine to uridine in the pyrimidine salvage pathway in *S. cerevisiae* [32]. In *A. gossypii*, in the pyrimidine salvage pathway, uracil phosphoribosyltransfrase, encoded by the *AgFUR1* gene, controls the amount of phosphoribosyl pyrophosphate (PRPP), which is one of the precursors of riboflavin in this organism [33].

Regarding the riboflavin production in *A. gossypii*, one missense homozygous mutation (1180G → A) was detected in *AgSOK2* gene (AGOS_ABR055C) of MT strain. AgSOK2 is one of fungal-specific group of transcription factors and involved in the sporulation and riboflavin production in *A. gossypii* [34]. Deletion of *AgSOK2* gene led to the strong reduction of the riboflavin production and the deficiency of the sporulation by the downregulation of *AgIME2* and *AgNDT80* gene. In MT strain, the riboflavin overproduction and the sporulation deficiency were observed even though *AgSOK2* gene had one homozygous mutation. Therefore, it is possible that the riboflavin production and the sporulation in *A. gossypii* may be regulated differently by AgSOK2 or the homozygous mutation in *AgSOK2* gene may cause the sporulation deficiency but may not cause the reduction of riboflavin production.

Two homozygous mutation (2317A → T and 2281A → C) in *AgOAF1* gene (AGOS_ADR404C) were also found in the genome of MT strain. In the conventional medium previously reported (initial rapeseed oil concentration 100 g/L) [13], WT and MT strains consumed 78.6 and 62.7 g/L of rapeseed oil for 144 and 168 h cultivation in a 3 L jar-fermentor, respectively (unpublished data). Riboflavin production in WT and MT strains during the cultivation was 1.52 and 6.49 g/L, respectively. This result corresponded to the data in this study showing two homozygous mutations in *AgOAF1* gene (AGOS_ADR404C) encoding a subunit of an oleate-activated transcription factor which binds to the oleate response element in promoters of oleate-responsive genes. *A. gossypii* has more two genes encoding homologs of ScOAF1 gene (AGOS_ADR403C and AGOS_ADR405C). AGOS_ADR403C and AGOS_ADR405C also had one and two heterozygous mutations, respectively (Supplementary material Table S2).

In the MT strain, 1377 heterozygous mutations in the coding sequences were also detected (Supplementary material Table S2). Heterozygous mutations usually lead to less critical effects than homozygous mutations [35, 36]. However, heterozygous mutations sometimes have negative effects on protein functions as well as haploinsufficiency [37, 38]. In addition, some mutated proteins that form multimers exhibit dominant-negative effects on functions [39, 40]. Therefore, it is possible that heterozygous mutations also have some effect on riboflavin production in the MT strain. Among the 1377 heterozygous mutations in the coding sequences, unusual heterozygous mutations were detected (Table 2). Most genes in the TCA cycle have heterozygous mutations. In particular, three genes, namely, *AgSDH1* (AGOS_ACR052W), *AgSDH2* (AGOS_ACL065C), and *AgSDH3* (AGOS_AFR207C), encoding subunits of succinate dehydrogenase in *S. cerevisiae*, have heterozygous mutations. In addition, several genes encoding flavoproteins in the mitochondria also have heterozygous mutations. AgSDH1 is also a flavoprotein. Flavoproteins in mitochondria of yeasts function in redox processes via the transfer of electrons [41]. In addition, the flavin in flavoproteins participates in the reduction of heme iron or iron-sulfur clusters. In this study, we detected several homozygous mutations (*AgARO2*, *AgILV2*) and heterozygous mutations {*AgSDH1*, *AgPDX1* (AGOS_AGR323C), *AgNDI1* (AGOS_AFR447C), *AgDLD1* (AGOS_AER321W), *AgCBR1* (AGOS_ADL087W), *AgGLR1* (AGOS_AGR196W), *AgMTO1* (AGOS_AGR196W), *AgMET5* (AGOS_ABL077W), *AgPUT1* (AGOS_AGL165W), *AgFAS1* (AGOS_AER085C), *AgHEM14* (AGOS_AAR021W), *AgERV2* (AGOS_ACR175W), and *AgERO1* (AGOS_ADL348W)} in genes encoding flavoproteins in *S. cerevisiae*. It is possible that the riboflavin overproduction in the MT strain is associated with these mutations of genes encoding flavoproteins and dysfunction of the TCA cycle. MT strain is hypothesized to have mitochondrial dysfunction because most genes in the TCA cycle and genes encoding flavoproteins have heterozygous mutations. One homozygous mutation in *AgILV2* gene which encodes a flavoprotein, AHAS, localized in mitochondria, was also found (Tables 2 and 3). In humans, riboflavin supplementation rescues the mitochondrial disorders associated with the deficiencies of some flavoproteins and respiratory chains [42]. Additionally, we previously reported that the expression of genes involved in TCA cycles in MT strain was decreased compared to WT strain. Also the MT strain shown the decreased succinate and increased lactate and pyruvate compared to WT strain [13, 16]. These previous results also suggest the overproduction of riboflavin in the MT strain may also be associated with mitochondrial dysfunction.

**Table 2:** Heterozygous mutations in genes involved in metabolisms

Chromosome	Position	Wt seq.	MT seq	Quality	Mutation	Gene	Product	DNA changes	Protein changes	Read number		MT seq. Ratio
										WT seq.	MT seq.	
Glycolysis/Gluconeogenesis												
III	456,890	C	T	327.19	missense	AGOS_ACR056W	Phosphoglycerate mutase (AgGPM1)	c.374C > T	p.A125V	28	12	0.300
IV	287,997	T	C	503.19	missense	AGOS_ADL237C	6-phosphofructo-2-kinase (AgPFK26)	c.1796A > G	p.D599G	24	18	0.429
IV	1,362,124	A	T	725.19	missense	AGOS_ADR368W	Pyruvate kinase (AgPYK1)	c.1040A > T	p.K347M	21	23	0.523
V	242,262	A	C	708.19	missense	AGOS_AEL208W	Alpha subunit of phosphofructokinase (AgPFK1)	c.2255A > C	p.K752T	27	23	0.460
V	426,255	C	T	700.19	missense	AGOS_AEL106W	Fructose-2,6-bisphosphatase (AgFBP26)	c.103C > T	p.R35W	21	23	0.523
VI	96,950	A	C	1088.19	missense	AGOS_AFL185W	Beta subunit of phosphofructokinase (AgPFK2)	c.1963A > C	p.N655H	35	37	0.514
VI	97,509	AT	A	907.15	frameshift	AGOS_AFL185W	Beta subunit of phosphofructokinase (AgPFK2)	c.2526Tdel	p.Phe842fs	20	32	0.615
TCA cycle												
I	346,384	G	A	758.19	missense	AGOS_AAR004C	Citrate synthase (AgCIT1)	c.68C > T	p.T23M	18	25	0.581
I	634,291	G	T	991.19	Nonsense	AGOS_AAR162C	Pyruvate carboxylase (AgPYC2)	c.3266c > A	p.S1089*	31	33	0.514
I	634,669	A	T	836.19	missense	AGOS_AAR162C	Pyruvate carboxylase (AgPYC2)	c.2888 T > A	p.L963Q	21	26	0.553
III	238,489	T	G	729.19	missense	AGOS_ACL065C	Iron-sulfur protein subunit of succinate dehydrogenase (AgSDH2)	c.697A > C	p.T233P	25	23	0.479
III	238,962	G	A	1051.19	missense	AGOS_ACL065C	Iron-sulfur protein subunit of succinate dehydrogenase (AgSDH2)	c.224C > T	p.T75M	20	32	0.615
III	451,903	G	A	879.19	missense	AGOS_ACR052W	Flavoprotein subunit of succinate dehydrogenase (AgSDH1)^a^	c.1132G > A	p.D378N	17	27	0.614
IV	403,968	C	T	488.19	missense	AGOS_ADL164C	Malate dehydrogenase (AgMDH2)	c.196G > A	p.A66T	27	16	0.372
IV	644,214	A	G	568.19	missense	AGOS_ADL032W	Aconitase (AgACO1)	c.1367A > G	p.D456G	10	16	0.615
V	1,328,889	C	A	922.19	missense	AGOS_AER374C	Subunit of the mitochondrial alpha-ketoglutarate dehydrogenase (AgKGD1)	c.1837G > T	p.D613Y	27	27	0.5
V	1,328,948	G	A	711.19	missense	AGOS_AER374C	Subunit of the mitochondrial alpha-ketoglutarate dehydrogenase (AgKGD1)	c.1778C > T	p.T593M	25	23	0.479
VI	810,404	G	T	482.19	missense	AGOS_AFR207C	Subunit of succinate dehydrogenase (AgSDH3)	c.200C > A	p.S67Y	20	18	0.473
VI	1,103,105	G	A	636.19	missense	AGOS_AFR367W	Fumarate reductase (AgOSM1)	c.622G > A	p.A208T	21	19	0.475
VI	1,585,840	G	T	635.19	missense	AGOS_AFR629W	Aconitase (AgACO2)	c.1894G > T	p.D632Y	36	24	0.400
VII	1,652,466	A	G	970.19	missense	AGOS_AGR323C	E3-binding protein of pyruvate dehydrogenase (AgPDX1) ^a^	c.677 T > C	p.L226P	16	28	0.636
VI	681,082	C	T	624.19	missense	AGOS_AFR134C	Alpha subunit of succinyl-CoA ligase (AgLSC1)	c.193G > A	p.A65T	23	24	0.510
Mitochondria												
II	324,797	A	G	633.19	missense	AGOS_ABL038W	Mitochondrial aspartate aminotransferase (AgAAT1)	c.224A > G	p.D75G	20	19	0.487
II	325,256	C	T	487.19	missense	AGOS_ABL038W	Mitochondrial aspartate aminotransferase (AgAAT1)	c.683C > T	p.T228M	25	18	0.419
IV	532,772	C	A	1079.19	missense	AGOS_ADL087W	Cytochrome b reductase (AgCBR1)^a^	c.155C > A	p.T52N	25	34	0.576
IV	1,458,400	G	T	559.19	missense	AGOS_ADR417W	Mitochondrial aldehyde dehydrogenase (AgALD4)	c.561G > T	p.W187C	21	17	0.447
V	1,227,029	G	A	503.19	missense	AGOS_AER321W	Mitochondrial D-lactate dehydrogenase (AgDLD1) ^a^	c.190G > A	p.A64T	11	15	0.577
VI	899,775	G	A	668.19	missense	AGOS_AFR255W	Mitochondrial tRNA translation optimization 1 (MTO1) ^a^	c.1423G > A	p.G475S	27	22	0.449
VI	1,243,899	C	T	819.19	missense	AGOS_AFR447C	NADH:ubiquinone oxidoreductase (AgNDI1) ^a^	c.943G > A	p.V315M	16	26	0.619
VII	1,441,269	C	A	874.19	missense	AGOS_AGR196W	Glutathione-disulfide reductase (AgGLR1) ^a^	c.1415C > A	p.S472Y	27	28	0.509
Riboflavin metabolism												
II	194,781	G	T	733.19	missense	AGOS_ABL109W	Riboflavin kinase (AgFMN1)	c.80G > T	p.S27I	20	22	0.524
IV	182,017	G	A	687.19	missense	AGOS_ADL296C	GTP cyclohydrolase II (AgRIB1)	c.230C > T	p.P77L	23	23	0.500
Glycine, serine, threonine metabolism												
I	448,391	G	A	962.19	missense	AGOS_AAR059C	Threonine synthase (AgTHR4)	c.685C > T	p.R229W	19	29	0.604
III	125,457	G	A	821.19	missense	AGOS_ACL130C	Phosphoserine phosphatase (AgSER2)	c.140C > T	p.A47V	28	27	0.491
III	727,688	C	T	572.19	missense	AGOS_ACR215C	Serine hydroxymethyltransferase (AgSHM2)	c.593G > A	p.R198Q	24	20	0.455
VII	1,057,290	T	C	592.19	missense	AGOS_AGR012C	Cystathionine beta-synthase (AgCYS4)	c.269A > G	p.K90R	16	19	0.543
VII	1,446,998	A	G	720.19	missense	AGOS_AGR200W	Threonine aldolase (AgGLY1)	c.1088A > G	p.Y363C	14	20	0.588
Branched-chain amino acid metabolism												
I	305,862	G	A	960.19	missense	AGOS_AAL021W	Small subunit of acetohydroxyacid synthase (AgILV6)	c.140G > A	p.S47N	25	29	0.537
I	305,877	G	A	923.19	missense	AGOS_AAL021W	Small subunit of acetohydroxyacid synthase (AgILV6)	c.155G > A	p.S52N	28	30	0.517
I	306,395	G	T	711.19	missense	AGOS_AAL021W	Small subunit of acetohydroxyacid synthase (AgILV6)	c.673G > T	p.G225C	23	22	0.489
II	729,493	G	A	1028.19	missense	AGOS_ABR174W	Branched-chain amino acid biosynthesis activator (AgLEU3)	c.704G > A	p.G235D	23	33	0.589
II	730,278	G	A	915.19	missense	AGOS_ABR174W	Branched-chain amino acid biosynthesis activator (AgLEU3)	c.1489G > A	p.A497T	22	26	0.542
VI	12,855	C	A	543.19	missense	AGOS_AFL229W	2-isopropylmalate synthase (AgLEU4)	c.1051C > A	p.P351T	26	19	0.422
VII	1,381,676	C	T	564.19	missense	AGOS_AGR169W	3-isopropylmalate dehydratase (LEU1)	c.226C > T	p.H76Y	12	17	0.586
VII	1,382,933	T	C	745.19	missense	AGOS_AGR169W	3-isopropylmalate dehydratase (LEU1)	c.1483 T > C	p.S495P	26	25	0.490
Aromatic amino acid metabolism												
II	206,627	C	T	580.19	missense	AGOS_ABL102C	3-deoxy-D-arabino-heptulosonate-7-phosphate (DAHP) synthase (AgARO3)	c.935G > A	p.C312Y	28	18	0.391
II	799,743	C	A	554.19	missense	AGOS_ABR209W	Anthranilate synthase (AgTRP2)	c.982C > A	p.L328I	15	16	0.516
VI	1,313,765	T	A	750.19	missense	AGOS_AFR485C	Tryptophan synthase (AgTRP5)	c.1917A > T	p.Q639H	33	29	0.468
VI	1,426,745	G	T	476.19	missense	AGOS_AFR548C	Aromatic aminotransferase I (AgARO8)	c.544C > A	p.P182T	29	16	0.356
VII	1,157,861	G	A	690.19	missense	AGOS_AGR066W	Pentafunctional aromatic polypeptide (AgARO1)	c.3536G > A	p.R1179H	22	21	0.488
VII	1,158,247	G	A	921.19	missense	AGOS_AGR066W	Pentafunctional aromatic polypeptide (AgARO1)	c.3922G > A	p.G1308S	29	31	0.517
Sulfur amino acid metabolism												
I	361,523	G	A	834.19	missense	AGOS_AAR010W	Transcriptional activator of sulfur metabolism (AgMET28)	c.719G > A	p.R240Q	28	25	0.472
II	259,309	C	T	578.19	missense	AGOS_ABL077W	Beta subunit of sulfite reductase (AgMET5) ^a^	c.3002C > T	p.A1001V	25	19	0.432
II	804,448	C	T	685.19	missense	AGOS_ABR212C	Cobalamin-independent methionine synthase (AgMET6)	c.499G > A	p.G167S	25	23	0.479
III	259,886	C	A	1053.19	missense	AGOS_ACL059C	Peroxisomal cystathionine beta-lyase (AgSTR3)	c.1210G > T	p.V404L	16	32	0.667
III	585,577	C	A	547.19	missense	AGOS_ACR134W	Folylpolyglutamate synthetase (AgMET7)	c.1135C > A	p.L379M	25	17	0.405
IV	646,485	G	A	838.19	missense	AGOS_ADL031W	O-acetyl homoserine-O-acetyl serine sulfhydrylase (AgMET17)	c.302G > A	p.G101D	23	26	0.531
V	1,338,633	G	A	974.19	missense	AGOS_AER377C	Component of cytosolic iron-sulfur protein assembly machinery (AgMET18)	c.1061C > T	p.T354I	28	31	0.525
VI	1,699,984	C	A	936.19	missense	AGOS_AFR682C	L-homoserine-O-acetyltransferase (AgMET2)	c.1045G > T	p.A349S	20	32	0.615
VI	1,720,007	C	T	393.19	missense	AGOS_AFR692C	S-adenosylmethionine synthetase (AgSAM2)	c.731G > A	p.G244D	30	13	0.302
VII	1,511,391	C	A	892.19	missense	AGOS_AGR237C	Alpha subunit of assimilatory sulfite reductase (AgMET10)	c.2268G > T	p.E756D	22	28	0.560
VII	1,512,792	C	A	606.19	missense	AGOS_AGR237C	Alpha subunit of assimilatory sulfite reductase (AgMET10)	c.867G > T	p.E289D	27	22	0.449
VII	1,685,571	G	A	715.19	missense	AGOS_AGR343W	Component of cytosolic iron-sulfur protein assembly (CIA) machinery	c.563G > A	p.R188H	24	21	0.467
Other amino acid metabolism												
III	169,882	C	T	367.19	missense	AGOS_ACL096W	Proline utilization transactivator (AgPUT3)	c.382C > T	p.R128W	26	14	0.350
IV	98,235	C	A	727.19	missense	AGOS_ADL346W	Alpha-aminoadipate reductase (AgLYS2)	c.1648C > A	p.L550M	27	23	0.460
VI	1,397,559	C	A	864.19	missense	AGOS_AFR534W	Small subunit of carbamoyl phosphate synthetase (AgCPA1)	c.976C > A	p.P326T	29	25	0.463
VII	389,521	C	A	724.19	missense	AGOS_AGL165W	Proline oxidase (AgPUT1) ^a^	c.104C > A	p.T35K	28	24	0.462
VII	1,708,538	T	G	528.19	missense	AGOS_AGR357W	Asparaginase (AgASP1)	c.311 T > G	p.I104R	19	17	0.472
Purine, pyrimidine nulceotide metabolism												
I	558,677	G	A	532.19	missense	AGOS_AAR120C	Phosphoribosyl-glycinamide transformylase (AgADE8)	c.218C > T	p.T73I	16	16	0.500
II	269,595	C	A	593.19	missense	AGOS_ABL070C	Xanthine-guanine phosphoribosyl transferase (AgXPT1)	c.232G > T	p.D78Y	29	22	0.431
II	784,947	G	A	618.19	missense	AGOS_ABR204C	AMP deaminase (AgAMD1)	c.1553C > T	p.T518I	38	21	0.356
III	132,857	C	A	773.19	missense	AGOS_ACL121C	Trifunctional C1-tetrahydrofolate synthase (AgADE3)	c.2067G > T	p.R689S	21	25	0.543
III	214,069	A	T	441.19	missense	AGOS_ACL077C	Ribose-5-phosphate isomerase (AgRKI1)	c.17 T > A	p.I6N	29	17	0.370
III	636,192	A	T	711.19	missense	AGOS_ACR160C	Nicotinate phosphoribosyltransferase (AgNPT1)	c.84 T > A	p.N28K	26	24	0.480
III	654,234	C	T	690.19	missense	AGOS_ACR170C	Uridylate kinase (AgURA6)	c.152G > A	p.R51H	14	21	0.600
III	715,325	G	T	697.19	missense	AGOS_ACR210C	Phosphoribosylaminoimidazole carboxylase (AgADE2)	C.926C > A	p.A309D	23	21	0.477
III	832,220	C	T	957.19	missense	AGOS_ACR263C	Bifunctional carbamoylphosphate synthetase/aspartate transcarbamylase (AgURA2)	c.2275G > A	p.E759K	19	31	0.620
III	832,428	C	T	745.19	missense	AGOS_ACR263C	Bifunctional carbamoylphosphate synthetase/aspartate transcarbamylase (AgURA2)	c.2067G > A	p.M689I	27	23	0.460
IV	580,072	G	T	461.19	missense	AGOS_ADL057W	Large subunit of ribonucleotide reductase (AgRNR1)	c.2520G > T	p.K840N	16	15	0.483
V	792,520	T	C	1069.19	missense	AGOS_AER083C	5-phospho-ribosyl-1-pyrophosphate synthetase (AgPRS1)	c.488A > G	p.Q163R	24	33	0.578
VI	896,312	A	T	502.19	missense	AGOS_AFR254C	Aminoimidazole ribotide synthetase and glycinamide ribotide synthetase (AgADE5,7)	c.1654 T > A	p.L552I	20	15	0.428
VI	978,821	C	A	584.19	missense	AGOS_AFR297W	Myb-related transcription factor (AgBAS1)	c.905C > A	p.P302H	23	20	0.465
VII	108,330	G	A	674.19	missense	AGOS_AGL320C	CTP synthase (AgURA7)	c.1361C > T	p.T454I	23	25	0.521
VII	430,379	G	A	703.19	missense	AGOS_AGL146W	GTP cyclohydrolase (AgURC1)	c.1247G > A	p.G416D	27	21	0.438
VII	1,072,826	T	C	377.19	missense	AGOS_AGR022C	Nicotinic acid mononucleotide adenylyltransferase (AgNMA1)	c.814A > G	p.T272A	26	13	0.333
Fatty acid metabolism												
I	564,702	G	A	769.19	missense	AGOS_AAR124C	Carnitine acetyl-CoA transferase (AgCAT2)	c.1736C > T	p.S579F	19	23	0.548
IV	1,430,996	G	A	829.19	missense	AGOS_ADR403C	Oleate-activated transcription factor (AgOAF1 or AgPIP2)	c.1405C > T	p.R469C	22	26	0.542
IV	1,436,329	G	T	757.19	missense	AGOS_ADR405C	Oleate-activated transcription factor (AgOAF1 or AgPIP2)	c.2170C > A	p.L724I	35	28	0.444
IV	1,437,793	G	A	800.19	stop_gained	AGOS_ADR405C	Oleate-activated transcription factor (AgOAF1 or AgPIP2)	c.706C > T	p.Q236*	30	25	0.455
IV	1,443,883	T	A	729.19	nonsense	AGOS_ADR408W	Acetyl-coA synthetase (AgACS1)	c.1128 T > A	p.Tyr376*	21	23	0.523
V	794,683	T	C	631.19	missense	AGOS_AER085C	Beta subunit of fatty acid synthetase (AgFAS1) ^a^	c.5837A > G	p.K1946R	23	19	0.452
V	797,843	T	G	419.19	missense	AGOS_AER085C	Beta subunit of fatty acid synthetase (AgFAS1) ^a^	c.2677A > C	p.K893Q	38	15	0.283
V	797,858	C	A	1299.19	missense	AGOS_AER085C	Beta subunit of fatty acid synthetase (AgFAS1) ^a^	c.2662G > T	p.D888Y	14	38	0.731
VI	172,719	A	T	746.19	missense	AGOS_AFL138W	Alpha subunit of fatty acid synthetase (AgFAS2)	c.7A > T	p.M3L	19	26	0.578
VI	175,856	C	A	656.19	missense	AGOS_AFL138W	Alpha subunit of fatty acid synthetase (AgFAS2)	c.3144C > A	p.F1048L	21	21	0.500
VI	1,507,650	T	A	742.19	missense	AGOS_AFR592W	1-acyl-sn-glycerol-3-phosphate acyltransferase (AgSLC1)	c.832A > T	p.L278M	21	22	0.512
VII	421,657	G	T	728.19	missense	AGOS_AGL148C	Acetyl-coA synthetase (AgACS2)	c.772C > A	p.Q258K	24	24	0.500
VII	422,089	C	T	804.19	missense	AGOS_AGL148C	Acetyl-coA synthetase (AgACS2)	c.340G > A	p.A114T	25	26	0.510
VII	913,244	C	A	553.19	missense	AGOS_AGL060W	3-hydroxyacyl-CoA dehydrogenase and enoyl-CoA hydratase (AgFOX2)	c.814C > A	p.P272T	25	17	0.405
Heme biosynthesis												
I	380,486	A	C	573.19	missense	AGOS_AAR021W	Protoporphyrinogen oxidase (AgHEM14) ^a^	c.617A > C	p.E206A	30	21	0.412
II	203,080	T	C	1002.19	missense	AGOS_ABL104C	5-aminolevulinate synthase (AgHEM1)	c.1397A > G	p.E466G	18	31	0.633
V	1,281,270	T	A	552.19	nonsense	AGOS_AER351W	Uroporphyrinogen-III synthase (AgHEM4)	c.762 T > A	p.Y254*	19	20	0.513
VII	1,608,654	A	AG	453.15	frameshift	AGOS_AGR298C	S-adenosyl-L-methionine uroporphyrinogen III transmethylase (AgMET1)	c.1412dupC	p.A472fs	19	19	0.500
Other flavoprotein												
III	660,436	G	T	853.19	missense	AGOS_ACR175W	Sulfhydryl oxidase (AgERV2) ^a^	c.441G > T	p.W147C	28	28	0.500
IV	94,090	G	A	485.19	missense	AGOS_ADL348W	Endoplasmic oxidoreductin1 (AgERO1) ^a^	c.386G > A	p.S129N	20	18	0.474
Folate metabolism												
VII	1,665,459	G	A	658.19	missense	AGOS_AGR330W	Aminodeoxychorismate lyase (AgABZ2)	c.208G > A	p.V70M	29	23	0.442
VII	1,674,504	C	A	813.19	missense	AGOS_AGR335C	GTP-cyclohydrolase I (AgFOL2)	c.343G > T	p.D115Y	30	25	0.455

These heterozygous mutations are a subset among all 1382 heterozygous mutations which are shown in Table S2

^a^Flavoproteins

*Translation stops here

**Table 3:** Number of mutated genes encoding flavoproteins

	Total^a^	Homozygous	Heterozygous	Mutation rate
FAD-dependent	36	1 (1)	11 (9)	33.3%
FMN-dependent	16	1 (1)	2 (1)	18.8%

^a^Total number of each flavoproteins is showed based on the reference by Gudipati et al. [42]

Each bracket indicates the number of mutated genes encoding mitochondrial proteins

Related to the heterozygous mutations in flavoprotein genes, a heterozygous mutation in the *AgFMN1* gene (AGOS_ABL109W) was detected (Table 2). In *S. cerevisiae*, this gene encodes riboflavin kinase, which catalyzes the synthesis of FMN from riboflavin. FMN is converted to FAD by FAD synthase. The downregulation of *AgFMN1* gene expression prevented riboflavin consumption in this fungus, and the *ribC*-deleted mutant deregulated riboflavin production in *B. subtilis* by preventing FMN and FAD accumulation [43, 44]. Therefore, this mutation may partially contribute to riboflavin overproduction in the MT strain by partial restriction of the riboflavin flow to FMN. Additionally, heterozygous mutations were also detected in genes involved in heme biosynthesis and sulfur metabolism (Table 2).

We detected homozygous mutations in the *AgSHM2*, *AgARO2*, *AgILV2*, and *AgLYS5* genes involved in amino acid biosynthesis (Table 1). Heterozygous mutations in genes involved in amino acid metabolism were concentrated in glycine, serine, and threonine metabolism; branched-chain amino acid biosynthesis; and aromatic amino acid biosynthesis (Table 2). In our previous study, the increased expression of AgTRP2 (ABR209W) and AgTRP5 (AFR485C) was observed in MT strain by a proteomic analysis. AgTRP2 and AgTRP5 are annotated as anthranilate synthase and tryptophan synthase, respectively, which belong to the tryptophan biosynthetic pathway. These results suggest that these amino acid metabolic pathways may be linked to riboflavin production in *A. gossypii*.

Several heterozygous mutations were detected in genes involved in sulfur amino acid metabolism. In particular, the sulfur amino acid biosynthesis pathway contains heterozygously mutated genes in the MT strain {*AgMET5* (AGOS_ABL077W), *AgMET6* (AGOS_ABR212C), *AgSTR3* (AGOS_ACL059C), *AgMET17* (AGOS_ADL031W), *AgMET2* (AGOS_AFR682C), *AgSAM2* (AGOS_AFR692C), *AgMET10* (AGOS_AGR237C)}. Mainly, genes encoding all enzymes that catalyze homocysteine in *S. cerevisiae*, except the adenosylhomocysteinase encoded by the *AgSAH1* gene, were heterozygously mutated. These results suggest that methionine metabolism, which consists of one-carbon metabolism together with folate metabolism, may be associated with riboflavin production in *A. gossypii*. The *AgMET10* and *AgMET5* genes encode alpha and beta subunits of sulfite reductase *in S. cerevisiae*, respectively, which are both flavoproteins.

It was previously reported that riboflavin production in *A. gossypii* was improved by disruption of the *AgURA3* gene, which leads to blockage of the pyrimidine biosynthetic pathway in this organism [33]. In the MT strain, several genes in the pyrimidine biosynthetic pathway have heterozygous mutations (Table 2). These results suggest that pyrimidine metabolism, including the pyrimidine de novo and salvage pathways, may be associated with riboflavin production in *A. gossypii*. In the purine biosynthetic pathway, the *AgRKI1* (AGOS_ACL077C), *AgRPS1* (AGOS_AER083C), *AgADE5,7* (AGOS_AFR254C), *AgADE8* (AGOS_AAR120C), and *AgADE2* (AGOS_ACR210C) genes have heterozygous mutations in the MT strain. Moreover, the AgBAS1 gene (AGOS_AFR297W), which encodes the transcription factor for regulation of the purine and glycine biosynthesis pathways in *A. gossypii* [45], also has one heterozygous mutation. These heterozygous mutations may partially force the restriction of purine biosynthesis, which is important for riboflavin production in *A. gossypii*. This limited purine biosynthesis in *A. gossypii* was also reported by Ledesma-Amaro et al., who showed the downregulation of purine biosynthesis during riboflavin production [43].

In addition to mutations in genes involved in metabolic pathways in *A. gossypii*, 17 heterozygous mutations in genes involved in DNA repair were detected (Table 4). In particular, genes involved in mismatch DNA repair {*AgMSH2* (AGOS_AAL093C), *AgMSH3* (AGOS_ADR168C), *AgMSH6* (AGOS_AGR116W), *AgMLH1* (AGOS_AFL199C), *AgMLH2* (AGOS_AFR226C), *AgMLH3* (AGOS_AAL093C), and *AgPMS1* (AGOS_AER421W)} were heterozygously mutated. These proteins function cooperatively to repair DNA mismatches in *S. cerevisiae.* Among MutS homologs, genes encoding AgMSH2, AgMSH3 and AgMSH6 had heterozygous mutations, but no mutation was detected in genes encoding AgMSH1, AgMSH4 and AgMSH5. ScMSH2, ScMSH3 and ScMSH6 of *S. cerevisiae* function to maintain nuclear genome stability [46]. In contrast, ScMSH1 functions in mitochondria, and ScMSH4 and ScMSH5 function during meiosis [47, 48]. These results suggest that the heterologous mutations in AgMSH2, AgMSH3 and AgMSH6 may compromise the DNA mismatch repair pathway and contribute to the maintenance of DNA mismatches and accumulation of heterologous mutations in the genome of *A. gossypii* during disparity mutagenesis and rapid evolution of *A. gossypii* to the riboflavin-overproducing mutant MT strain. Previous papers have shown that heterologous mutations of the ScMSH2 gene showed mutator phenotypes in diploid yeasts and suppression of the mismatch repair pathway and proofreading-deficient DNA polymerase ε in human cells, leading to the accumulation of numerous mutations [49, 50]. However, the riboflavin production level in MT was stable during 14 passages [13].

**Table 4:** Heterozygous mutations in genes involved in DNA repair

Chromosome	Position	WT seq.	MT seq	Quality	Mutation	Gene	Product	DNA changes	Protein changes	Read number		MT seq ratio
										WT seq.	MT seq.	
I	177,825	G	T	533.19	missense	AGOS_AAL093C	DNA mismatch repair protein (AgMLH3)	c.1516C > A	p.L506M	22	17	0.436
IV	997,942	T	G	729.19	missense	AGOS_ADR168C	DNA mismatch repair protein (AgMSH3)	c.2937A > C	p.K979N	39	23	0.371
IV	998,607	G	A	982.19	nonsense	AGOS_ADR168C	DNA mismatch repair protein (AgMSH3)	c.2272C > T	p.Q758*	22	29	0.569
IV	1,446,658	G	T	747.19	missense	AGOS_ADR411W	Checkpoint protein (AgRAD17)	c.358G > T	p.D120Y	32	24	0.429
V	486,710	G	A	711.19	missense	AGOS_AEL075W	DNA polymerase delta subunit 3 (AgPOL32)	c.490G > A	p.A164T	18	22	0.550
V	1,239,357	C	A	411.19	missense	AGOS_AER327C	Uracil-DNA glycosylase (AgUNG1)	c.757G > T	p.A253S	27	16	0.372
V	1,445,972	G	A	869.19	missense	AGOS_AER421W	DNA mismatch repair protein (AgPMS1)	c.1762G > A	p.A588T	23	28	0.549
VI	65,368	C	T	587.19	missense	AGOS_AFL199C	DNA mismatch repair protein (AgMLH1)	c.320G > A	p.C107Y	20	20	0.500
VI	677,447	G	A	967.19	nonsense	AGOS_AFR133C	single-stranded DNA endonuclease (AgRAD2)	c.2143C > T	p.Q715*	25	30	0.545
VI	677,525	C	A	743.19	missense	AGOS_AFR133C	single-stranded DNA endonuclease (AgRAD2)	c.2065G > T	p.D689Y	27	28	0.509
VI	834,113	A	G	889.19	missense	AGOS_AFR220W	DNA helicase/Ubiquitin ligase (AgRAD5)	c.2419A > G	p.S807G	18	25	0.581
VI	834,860	G	T	678.19	nonsense	AGOS_AFR220W	DNA helicase/Ubiquitin ligase (AgRAD5)	c.3166G > T	p.E1056*	29	22	0.431
VI	848,262	A	G	828.19	missense	AGOS_AFR226C	DNA mismatch repair protein (AgMLH2)	c.1882 T > C	p.F628L	26	31	0.544
VI	1,528,970	C	T	1142.19	nonsense	AGOS_AFR603C	DNA mismatch repair protein (AgMSH2)	c.2711G > A	p.W904*	18	36	0.667
VI	1,529,553	G	A	1151.19	missense	AGOS_AFR603C	DNA mismatch repair protein (AgMSH2)	c.2128C > T	p.P710S	12	33	0.733
VII	1,278,725	T	G	786.19	missense	AGOS_AGR116W	DNA mismatch repair protein (AgMSH6)	c.1005 T > G	p.N335K	24	25	0.510
VII	1,368,167	C	T	788.19	missense	AGOS_AGR162C	DNA repair protein (AgRAD4)	c.1214G > A	p.R405Q	17	23	0.575

*Translation stops here

As mentioned above, MT strain never produced its haploid spores. Some heterozygous mutations were found in genes involved in the sporulation (Table 5). Two putative 1,3-β-D-glucan synthase genes (AGOS_ACL181C, AGOS_AAR053W) had heterozygous mutations. Especially, AGOS_AAR053W had one frameshift mutation which may have great influences on the protein function. In *S. cerevisiae*, FKS2 is a 1,3-β-D-glucan synthase during its sporulation and FKS2 and FKS3 works in spore wall assembly [51]. In addition, FKS2 binds to a sporulation-specific kinase, SMK1 [52]. Heterozygous mutations of AGOS_ACL181C and AGOS_AAR053W may have some influences on the sporulation in MT strain. Moreover, we found heterozygous mutations in *AgIME2* (AGOS_AFR076W) and *AgKAR4* (AGOS_AFR736C) genes. Disruption of *AgIME2* gene or *AgKAR4* gene leads to the deficiency of its sporulation in *A. gossypii* [53]. These heterozygous mutations may also be one of the reasons for the sporulation deficiency in MT strain.

**Table 5:** Heterozygous mutations in genes involved in sporulation

Chromosome	Position	WT seq.	MT seq	Quality	Mutation	Gene	Product	DNA changes	Protein changes	Read number		MT seq ratio
										WT seq.	MT seq.	
III	41,510	T	G	1253.19	missense	AGOS_ACL181C	1,3-beta-D-glucan synthase (AgFKS1 or AgGSC2)	c.4596A > C	p.Lys1532Asn	23	37	0.617
II	101,053	T	G	878.19	missense	AGOS_ABL159W	Component of the septin ring (AgSHS1)	c.1229 T > G	p.Ile410Ser	20	29	0.592
IV	649,360	G	A	886.19	missense	AGOS_ADL029W	Component of the meiotic outer plaque of the spindle pole body (AgSPO74)	c.374G > A	p.Ser125Asn	19	26	0.578
V	965,295	A	ACAG	1023.15	disruptive_inframe_insertion	AGOS_AER177W	Transcription factor targeting filamentation genes (AgTEC1)	c.1518_1520dupGCA	p.Gln507dup	21	28	0.571
IV	1,263,702	G	T	982.19	missense	AGOS_ADR317C	Dual-specificity kinase (AgMPS1)	c.2228C > A	p.Thr743Asn	24	31	0.564
VI	1,158,992	A	G	1022.19	missense	AGOS_AFR400C	N-formyltyrosine oxidase (AgDIT2)	c.635 T > C	p.Ile212Thr	25	32	0.561
III	48,243	C	A	765.19	missense	AGOS_ACL179C	Meiosis-specific protein (AgSPO77)	c.1601G > T	p.Arg534Ile	18	23	0.561
VI	1,225,184	A	C	753.19	missense	AGOS_AFR436C	Component of the septin ring (AgCDC11)	c.371 T > G	p.Val124Gly	21	25	0.543
I	436,519	A	AT	935.15	frameshift	AGOS_AAR053W	1,3-beta-D-glucan synthase (AgGSC2 or AgFKS1 or AgFKS3)	c.916_917insT	p.Arg306fs	24	28	0.538
VI	1,531,918	T	C	659.19	missense	AGOS_AFR604C	Component of the meiotic outer plaque of the spindle pole body (AgSPO21)	c.2531A > G	p.Gln844Arg	18	20	0.526
VI	1,288,694	G	T	943.19	missense	AGOS_AFR469W	t-SNARE protein (AgSEC9)	c.918G > T	p.Glu306Asp	27	29	0.518
VI	672,483	C	T	645.19	missense	AGOS_AFR130W	Protein involved in the control of meiotic nuclear division (AgSSP1)	c.121C > T	p.Leu41Phe	20	21	0.512
IV	1,456,401	G	T	500.19	missense	AGOS_ADR416W	Mitotic exit network scaffold protein (AgNUD1)	c.1263G > T	p.Gln421His	16	16	0.500
VI	639,702	C	T	610.19	missense	AGOS_AFR111C	Component of the septin ring (AgCDC3)	c.203G > A	p.Gly68Asp	22	22	0.500
VII	1,087,176	G	A	676.19	missense	AGOS_AGR031W	Transcriptional repressor (AgNRG1 or AgNRG2)	c.107G > A	p.Ser36Asn	25	24	0.490
VI	1,225,449	A	T	596.19	missense	AGOS_AFR436C	Component of the septin ring (AgCDC11)	c.106 T > A	p.Ser36Thr	25	21	0.457
V	1,436,486	G	A	466.19	missense	AGOS_AER416C	EH domain-containing protein (AgEND3)	c.4C > T	p.Pro2Ser	21	17	0.447
VI	566,815	A	C	758.19	missense	AGOS_AFR076W	Serine/threonine protein kinase (AgIME2)	c.1142A > C	p.Tyr381Ser	30	24	0.444
IV	1,423,888	G	A	643.19	missense	AGOS_ADR400W	Gamma-tubulin small complex receptor (AgSPC72)	c.278G > A	p.Ser93Asn	24	19	0.442
IV	650,376	A	T	523.19	missense	AGOS_ADL029W	Component of the meiotic outer plaque of the spindle pole body (AgSPO74)	c.1390A > T	p.Ile464Phe	26	19	0.422
VII	394,750	G	A	560.19	missense	AGOS_AGL162C	Sm-like protein (AgSEC1)	c.1972C > T	p.Pro658Ser	26	19	0.422
III	506,687	T	G	578.19	missense	AGOS_ACR083C	Meiosis-specific component of the spindle pole body (AgDON1 or AgCUE5)	c.237A > C	p.Arg79Ser	28	18	0.391
III	38,740	C	A	576.19	missense	AGOS_ACL182C	1,3-beta-glucanosyltransferase (AgGAS2)	c.1161G > T	p.Glu387Asp	40	21	0.344
VI	1,794,575	A	C	577.19	missense	AGOS_AFR736C	Transcription factor required for response to pheromones (AgKAR4)	c.422 T > G	p.Phe141Cys	46	21	0.313

Gene Ontology (GO) enrichment analysis was performed (Supplementary materials Tables S3, S4 and S5) in the set of genes containing homozygous or heterozygous mutations. Over-represented GO terms are ATP binding, Protein binding and ATPase activity. Especially, in “ATP binding”, all 22 ATP-dependent helicase genes have a single heterologous mutation, respectively. It was recently reported that RNA helicases have the relationship with aging and life span of cells [54]. Mutations of all RNA helicase genes support the suggestion that riboflavin production in *A. gossypii* may be associated with the aging of cells. Interestingly, we also found 25 mutated genes among 139 genes in “oxidation-reduction process” (Supplementary materials Table S3) and no mutated gene was in “mitochondrion”. This result suggests that oxidative stress is more associated with the riboflavin over-production in MT strain than the mitochondrial dysfunction and supports the previous study showing a riboflavin-overproducing *A. gossypii* mutant is vulnerable to photoinduced oxidative DNA damage and accumulate ROS [23], leading to the aging of cells. On the other hand, “Ribosome”, “Translation”, “Structural constituent of ribosome” and “Intracellular” were under-represented. These GO terms contain ribosomal proteins involved in translation (Supplementary materials Tables S5). Mutations of genes encoding these proteins are lethal in organisms and, therefore, these GO terms were under-presented.

### Effect of temperature on riboflavin production in MT strain

**Fig. 3:**
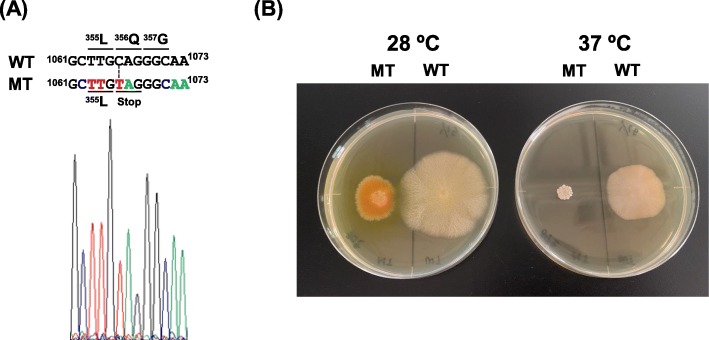
Growth and riboflavin production of MT strain. **a** Sequence of *AgHSP104* gene in the MT strain. The gene sequence was confirmed by Sanger method. **b** Growth of and riboflavin production in WT and MT on YD medium at 28 and 37 °C for 5 d

By genomic analysis of the MT strain, one homozygous mutation in the *AgHSP104* gene (AGOS_AGL036C), which causes a nonsense mutation, was detected (Table 1). This mutation generates the mutated AgHSP104, composed of 355 amino acid residues at its N-terminus. HSP104 in fungi contributes to the thermotolerance and disaggregation of denatured and aggregated proteins, ethanol tolerance and survival in the stationary phase [55]. We confirmed this nonsense mutation in the MT strain by DNA sequencing (Fig. 3a). In addition, other four homozygous mutations in the MT strain were also confirmed by DNA sequencing (Data not shown). These results validate the results of the genomic analysis. The WT and MT strains were cultivated on YD medium at 28 and 37 °C. The growth and riboflavin production in WT cultivated at 37 °C were slightly lower than those in WT cultivated at 28 °C (Fig. 3b). However, the growth of and riboflavin production in the MT strain were dramatically reduced at 37 °C compared to those at 30 °C, and the MT strain was not able to grow normally. These results reflected the generation of truncated AgHSP104 in the MT strain, leading to loss of thermotolerance, even at 37 °C. This result also confirms the presence of the homozygous mutation in the *AgHSP104* gene of the MT strain. Which corresponds, a homozygous missense mutation was found in *AgPMT1* gene (AGOS_ADR279C) (Supplementary materials Table S1). This encodes a putative *O*-mannosyltransferase which is essential for the cell wall integrity by *O*-glycosylation of cell wall mannoproteins. In *Aspergillus*, the disruption of the genes caused the high sensitivity of growth temperature and low cell wall integrity [56, 57]. This mutation may also partially contribute to the high sensitivity of growth temperature in MT strain.

### Effect of iron for the riboflavin production in MT strain

**Fig. 4:**
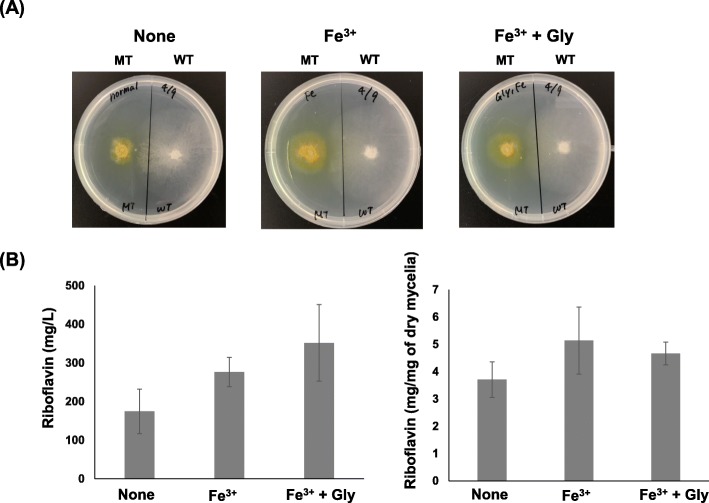
Growth and riboflavin production in the WT and MT strains in the presence of Fe^3+^ and glycine. **a** Growth of WT and MT strains on the minimum medium plate containing Fe^3+^ and glycine. Fe^3+^ and glycine were supplemented at 27 mg/L and 1 mM, respectively. **b** Riboflavin production of MT strain n minimal medium supplemented with 27 mg/L Fe^3+^ and 1 mM glycine. The amount of riboflavin and dry cell was measured at 4 days. Metal ions except for Fe^3+^ were not supplemented in both cultivations

In Tables 2 and 3, many heterozygous mutations were detected in genes encoding proteins involved in mitochondrial function and DNA. Iron-sulfur (Fe/S) clusters are required for TCA cycles, the electron transfer chain and fatty acid oxidation in mitochondria and DNA repair in nucleus [58, 59]. Therefore, the addition of iron ion for the MT strain cultivation was investigated. Fe^3+^ enhanced the growth of mycelia and riboflavin production in the MT strain (Fig. 4a) also in the presence of glycine, which is well-known for the improvement of the riboflavin production in *A. gossypii*. Addition of Fe^3+^ and Fe^3+^ + glycine improved the riboflavin production of MT strain by 1.6 and 2.0 fold, respectively although we were not able to find its significant differences. (Fig. 4b). Specific riboflavin production of MT strain in the presence of Fe^3+^ and Fe^3+^ + glycine were also improved by 1.4 and 1.3 fold, respectively although we were not able to find its significant differences. These results indicate that Fe^3+^ and glycine enhanced the riboflavin production by the improvement of its growth. Flavoproteins in mitochondria of yeasts function in redox processes via the transfer of electrons [41]. In addition, the flavin in flavoproteins participates in iron metabolism. We found two homozygous mutations (*AgARO2*, *AgILV2*) and 13 heterozygous mutations (*AgSDH1*, *AgPDX1*, *AgNDI1*, *AgDLD1*, *AgCBR1*, *AgGLR1*, *AgMTO1*, *AgMET5*, *AgPUT1*, *AgFAS1*, *AgHEM14*, *AgERV2*, and *AgERO1*) in genes encoding putative flavoproteins. Most of these flavoproteins may localized in mitochondria (Tables 1, 2 and 3). We previously reported that lactate and pyruvate was produced more in MT strain than WT strain in the minimum medium and succinate was decreased in MT strain compared to WT stain [16]. In addition, gene expression of most of genes involved in TCA cycle was down-regulated in MT strain cultivated compared to WT stain [13]. In Fig. 4, the growth and riboflavin production in MT strain were enhanced by the addition of iron ion, which is involved in mitochondrial functions with flavoproteins [41, 58, 59]. This result also supports the relationship of riboflavin production with the mitochondrial dysfunction. The addition of Fe^2+^ had no effect on the riboflavin production in WT strain (Data not shown).

## Conclusion

In this study, we analyzed the genomic sequence of the riboflavin-overproducing mutant MT strain and detected some intriguing homozygous and heterozygous mutations in the coding sequences of the MT genome. The homozygous and heterozygous mutations were concentrated in genes encoding proteins involved in the TCA cycle, mitochondrial functions, sulfur metabolism and DNA mismatch repair. The discovery of many heterozygous mutations indicates that mutants with many heterozygous mutations cannot be isolated by conventional mutagenesis methods, such as the use of mutagens and genetic engineering. Disparity mutagenesis is a promising tool for the creation of new types of eukaryotic mutants in various research fields and manufacturing industries. Additionally, the genomic analysis and GO enrichment analysis showed the relationship of the riboflavin production in MT strain with oxidative stress and the aging of cells, supporting the previous result that the accumulation of ROS and DNA damages appeared in other *A.gossypii* riboflavin-overproducing mutant [23].

## Methods

### Strains and cultivation

*A. gossypii* ATCC10895, which was purchased from American Type Culture Collection (ATCC), was used as a wild-type strain (WT strain). The *A. gossypii* w122032 mutant (MT strain) was previously isolated by disparity mutagenesis in the presence of H_2_O_2_, itaconate and oxalate [13] and used as a mutant strain in this study. These strains were maintained at 28 °C in YD medium (1% yeast extract, 1% glucose, pH 6.8). Chemically defined medium (15 g/L glucose as a carbon source, 1.5 g/L asparagine, 0.75 g/L KH_2_PO_4_, 0.1 g/L myo-inositol, pH 6.8) was used as a minimal medium [16]. To cultivate *A. gossypii* in flasks, mineral ions (4.4 mg/L CoCl_2_·6H_2_O, 18.0 mg/L MnCl_2_·4H_2_O, 44.0 mg/L ZnSO_4_·7H_2_O, 10.1 mg/L MgSO_4_·7H_2_O, 27.0 mg/L FeCl_3_·6H_2_O, 21.9 mg/L CaCl_2_·6H_2_O, and 2.7 mg/L CuSO_4_·5H_2_O) were added to the minimal medium. Cultivation was carried out using a 500-ml flask (working volume 50 ml) with an agitation rate of 120 rpm at 28 °C. The chemically defined medium was used for cultivation on agar plates. Each amino acid was used to supplement the media at 1 mM.

### Assay

The amount of riboflavin was determined according to a previous protocol [16]. Briefly, 0.8 mL of the culture broth was thoroughly mixed with 0.2 mL of 1 N NaOH. A 0.4-mL aliquot of the resulting solution was neutralized with 1 mL of 0.1 M potassium phosphate buffer (pH 6.0), and the absorbance of the solution at a wavelength of 444 nm was measured. The riboflavin concentration was calculated with an extinction coefficient of 1.04 × 10^− 2^ M^− 1^ cm^− 1^ (127 mg riboflavin/L at ABS444).

### Genome analysis

Genomic DNA was extracted from mycelia cultivated in YD medium during the logarithmic phase using the DNeasy Plant Mini Kit (Qiagen, Venlo, Netherlands) and fragmented using a Covaris Acoustic Solubilizer (Covaris, Woburn, MA, USA). Genomic libraries were prepared using the TruSeq Nano DNA Library Prep Kit (Illumina, San Diego, CA, USA) and sequenced using a MiSeq system (Illumina) at the Instrumental Research Support Office, Research Institute of Green Science and Technology, Shizuoka University.

Paired-end reads (2 × 301 bp) were cleaned up using Trimmomatic ver. 0.36 [60] by trimming adapter sequences, low-quality reads (quality score, < 15), and the final 301 bases, followed by filtering reads less than 150 bp. High-quality reads were aligned to the reference genome of *A. gossypii* ATCC10895 using BWA-MEM ver. 0.7.12 [61]. Aligned reads were sorted and duplicates were marked using Picard Tools ver. 2.8.0 (http://broadinstitute.github.io/picard/). The Genome Analysis Toolkit ver. 3.7 [62] was used to call variants, SNPs and short insertions/deletions (indels). The variants identified by HaplotypeCaller in GATK were filtered using Variant Filtration under the following settings: QualByDepth (QD) < 6.0; RMSMappingQuality (MQ) < 50; Quality (QUAL) < 100. Annotation of each variant and its functional effect was predicted using SnpEff ver. 4.3 T [63] with the default database of “*Ashbya_gossypii*”. All proteins of *A. gossypii* were annotated using HMMER 3.1b2 (http://hmmer.org) against Pfam database 32.0 [64]. GO terms associated with Pfam entries were assigned using the pfam2go mapping file (http://www.geneontology.org/external2go/pfam2go, version date of 2019/06/01). Two-sided Fisher’s exact test was performed to find the GO terms over- and under-represented in the homozygously and heterozygously mutated genes. The significance threshold of over- and under-represented GO terms was defined as a false discovery rate (FDR) of 0.05.

## Supplementary information

**Additional file 1: Table S1.** All 33 homozygous mutations detected in the coding sequences of the MT genome. **Table S2.** All 1377 heterozygous mutations detected in coding sequences of the MT genome. **Table S3.** Gene Ontology (GO) enrichment analysis of the genes containing mutations. **Table S4.** Genes assigned over-represented Gene Ontology. **Table S5.** Genes assigned under-represented Gene Ontology.

## Data Availability

The raw reads for *A. gossypii* strain WT and MT have been deposited in the DDBJ Sequence Read Archive (DRA) under the accession no. DRA008709. Additionally, they can be also accessed via NCBI (https://www.ncbi.nlm.nih.gov/sra/?term=DRA008709).

## Abbreviations

AHAS: Acetohydroxyacid synthase
Chr: Chromosome
FAD: Flavin adenine dinucleotide
FDR: False discovery rate
FMN: Favin mononucleotide
GO: Gene ontology
ICL: Isocitrate lyase
indels: insertions/deletions
MQ: RMSMappingQuality
ORF: Open reading frame
PPTase: 4′-Phosphopantetheinyl transferase
PRPP: Phosphoribosyl pyrophosphate
QD: QualByDepth
QUAL: Quality
ROS: Reactive oxygen species
SHMT: Serine hydroxymethyltransferase
SNP: Single-nucleotide polymorphisms
SNV: Single-nucleotide variant
